# Human Fragmentation Effects Are Genetically Detectable After 6 Years in an Island‐Endemic Plant

**DOI:** 10.1002/ece3.71310

**Published:** 2025-05-12

**Authors:** Wen‐Ting Jin, Shao‐Jun Ling, Myong Gi Chung, Mi Yoon Chung, Jordi López‐Pujol, Ming‐Xun Ren

**Affiliations:** ^1^ International Joint Center for Terrestrial Biodiversity Around South China Sea of Hainan Province, School of Ecology Hainan University Haikou China; ^2^ Center for Eco‐Environment Restoration Engineering of Hainan Province Hainan University Haikou China; ^3^ Division of Life Science and RINS Gyeongsang National University Jinju South Korea; ^4^ Department of Biological Sciences Chungnam National University Daejeon South Korea; ^5^ Botanic Institute of Barcelona (IBB) CSIC‐CMCNB Barcelona Spain; ^6^ Escuela de Ciencias Ambientales Universidad Espíritu Santo (UEES) Samborondón Ecuador

**Keywords:** anthropogenic disturbance, gene flow, Hainan Island, island biogeography

## Abstract

Anthropogenic disturbances have long been acknowledged to be one of the primary threats to biodiversity worldwide; however, little is still understood about how human‐built infrastructure affects gene flow and phylogeographic structure of plants. Such information is helpful for the conservation and restoration of human‐disturbed ecosystems. Here we studied the effects of a large reservoir and two expressways on *Primulina heterotricha* (Gesneriaceae), a short‐lived herb endemic to Hainan Island (China), one of the key areas of the globally important Indo‐Burma biodiversity hotspot. By applying comparative phylogeography using one nuclear ribosomal DNA and two chloroplast DNA sequences, we estimated the levels of genetic diversity and differentiation in 176 and 117 individuals collected, respectively, before (in 2016) and after (in 2022) the construction of two expressways in Hainan Island, from the same eight populations of *P. heterotricha*. We found that this species significantly increased nuclear genetic differentiation during the period 2016–2022, which coincides with the opening of the two expressways. Also notably, the sharing of ribotypes among the three groups of populations separated by the expressway network diminished greatly for the same period. Moreover, the changes in the significance of genetic barriers before and after road construction suggest that geographic isolation caused by human constructions is key for understanding the present phylogeographical patterns of *P. heterotricha*. We provide direct evidence that large anthropogenic infrastructures are capable of increasing genetic differentiation and, thus, modifying the phylogeographical pattern of *P. heterotricha*, in just a six‐year period (or two generations of the study plant). We suggest establishing ecological corridors to enhance gene exchange between the two sides of these artificial barriers.

## Graphical abstract

We provide direct evidence that anthropogenic infrastructures such as reservoirs and expressways have been capable of increasing genetic differentiation and, thus, modifying the phylogeographical pattern of *Primulina heterotricha* in just a six‐year period (two generations of the plant).

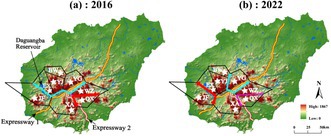

## 1 Introduction

Human activities, which are regarded as primary threats to biodiversity worldwide, are capable of disturbing habitat integrity and, thus, modifying phylogeographic patterns of naturally distributed species (Young et al. 1996; Aguilar et al. 2006; Martínez‐Ramos et al. 2016). For example, anthropogenic infrastructures such as expressways, buildings, and expansive farmlands can interrupt plant pollination and seed dispersal by hindering the movements of insects and other animals (Honnay and Jacquemyn 2007). Fragmentation of natural populations and geographical isolation of individuals may also lead to increased levels of inbreeding and genetic drift (Lowe et al. 2015), putting populations at risk of extinction (Tambarussi et al. 2017; Moraes et al. 2018).

**FIGURE 1:**
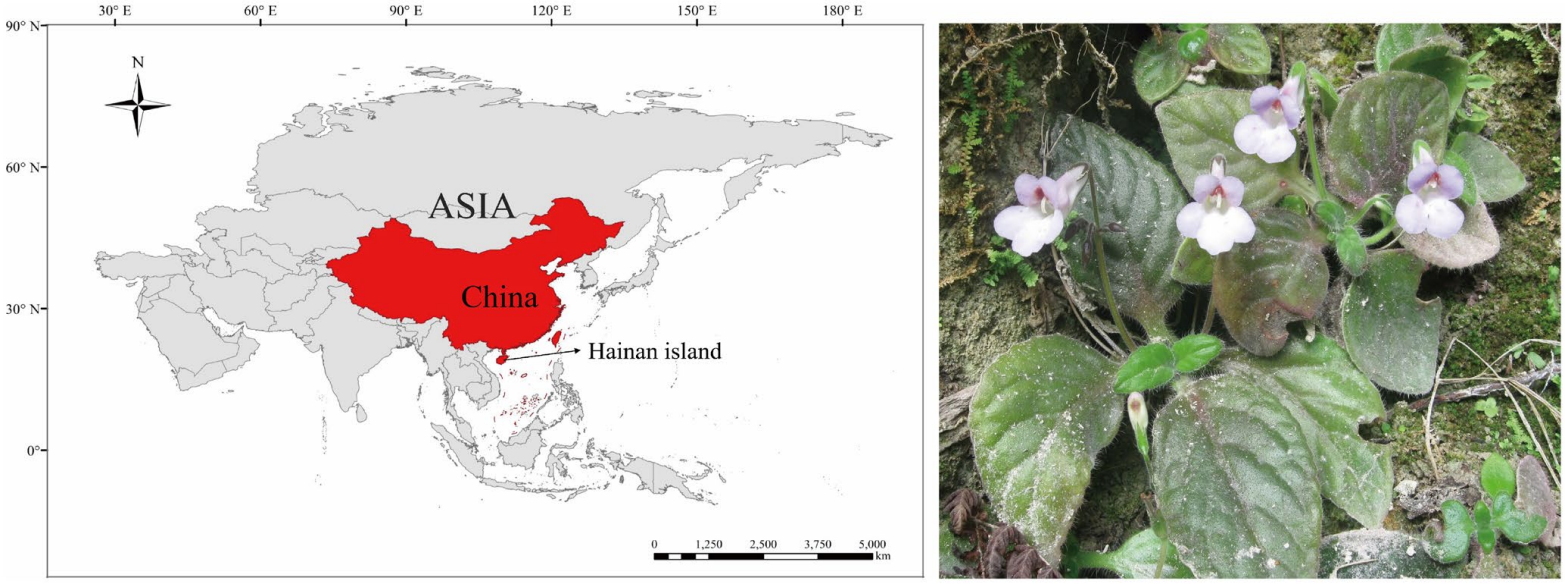
Location map of Hainan Island in China and flowering individuals of *Primulina heterotricha* (Gesneriaceae).

Hainan Island, located in South China (Figure 1), has an area of 35,000 km^2^ and is a distinctive part of the globally important Indo‐Burma biodiversity hotspot (Myers et al. 2000), as it harbors *ca*. 4800 vascular plants in total with nearly 500 endemics (Francisco‐Ortega et al. 2010a, 2010b; Yang 2013). Most of this rich plant diversity (e.g., 80% of the endemic species) is concentrated in the south‐central mountain system of the island (Francisco‐Ortega et al. 2010a, 2010b). Notably, this area is the home to the world's most endangered primate *Nomascus hainanus* (Long et al. 2021), highlighting its conservation value.

Currently, however, Hainan Island is experiencing a rapid economic development, which is partly based on the construction of large infrastructures; for example, the local authorities are aiming to build the world's biggest free trade port (www.xinhuanet.com/english/2018‐04/13/c_137109243.htm). The landscapes and vegetation have been severely affected during the most recent decades due to the accelerated growth of the human population and urbanization. For example, the Daguangba Reservoir on the Changhua River, the second largest river on the island, was completed in 1994 and formed a sluggish waterbody of nearly 6 km in width. This reservoir, located between Mt. E'xian and Mt. Jianfeng, may disrupt the connectivity of the natural vegetation of these two mountains. Moreover, two expressways were recently built: Road G9811 (hereafter referred to as Expressway 1), which was completed in 2018 and has a width of 26 m, and Road S10 (hereafter referred to as Expressway 2), built in 2019 and with a width of 20 m (www.xinhuanet.com/english/2021‐04/15/c_139882827.htm). These anthropogenic constructions lie in the middle part of the south‐central mountains on the island and may affect habitat integrity and the current population dynamics of the rare and endangered plants occurring in this part of the island, which may further result in changes in their phylogeographical structure.

The family Gesneriaceae on Hainan Island is characterized by its high levels of species diversity and endemism (Wei 2010; Ling et al. 2017a, 2017b). Fourteen genera and 25 species of the family occur on the island, including one endemic genus and 10 endemic species (Li and Wang 2005; Yang 2013; Ling et al. 2017a; Ling, Guan, et al. 2020). *Primulina heterotricha* (Merr.) Y. Dong & Yin Z. Wang (Figure 1), one of these endemic species, is widely distributed in the south‐central mountains on the island (Ling et al. 2017a, 2017b), which may be due, among other reasons, to its ability to grow in both acid and basic soils (Ai et al. 2015) The plant, a short‐lived herb (with a generation time of 1–3 years), has zygomorphic tubular flowers that are pollinated by several insects, particularly *Glossamegilla malaccensis* and *G. yunnanensis* of Anthophoridae (Ling et al. 2017a, 2017b). The fruit (capsule) is erect with small brown and fusiform‐shaped seeds, suggesting a poor dispersal capability that is likely associated with water courses (MXR, personal observations). *Primulina heterotricha* is a diploid plant with 2*n* = 36 (Kang et al. 2014). Taking all the above‐mentioned factors into consideration (i.e., its rather continuous distribution in the study area, its short generation time, and its restricted dispersal capabilities), this species is a good model system to uncover possible short‐term fragmentation effects of the Daguangba Reservoir and the newly built expressways on its genetic differentiation and phylogeographical patterns.

In this study, we addressed the following questions: (1) Are the recently built reservoir and expressways (i.e., artificial barriers) already influencing the genetic make‐up in the Hainan‐endemic *P. heterotricha*? and (2) if yes, how are these artificial barriers affecting the population differentiation and phylogeographical patterns of the study species? To get insights into these questions, we performed a population genetics and a phylogeographical study based on samples of *P. heterotricha* that were collected in 2016 and 2022 (i.e., before and after expressway construction), using conserved and slowly evolving nuclear and chloroplast DNA sequences.

## 2 Materials and Methods

### 2.1 Sample Collection and Laboratory Procedures

**FIGURE 2:**
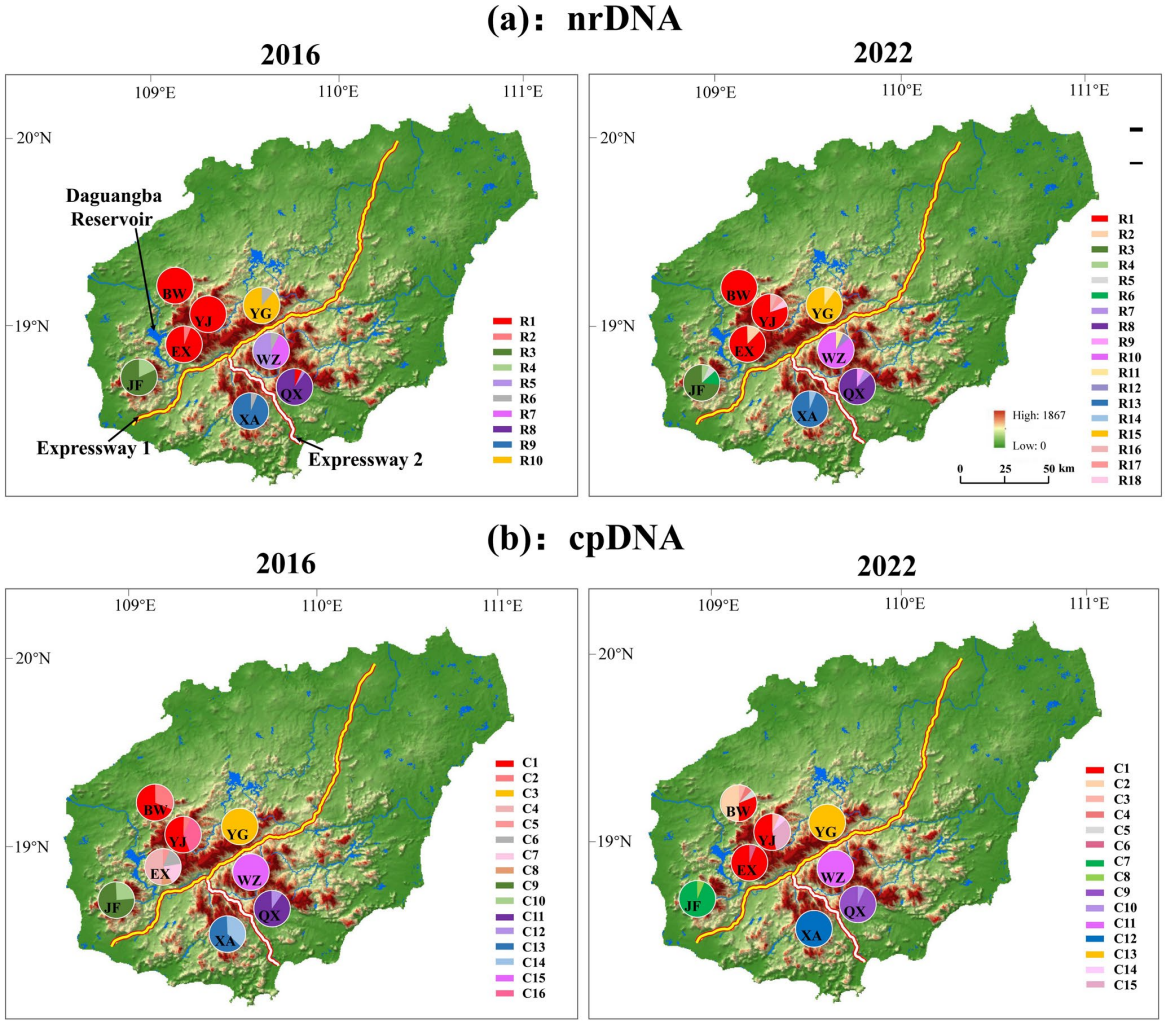
Haplotype distribution of (a) nrDNA ITS and (b) cpDNA *trn*L‐F and *ycf1b* in 2016 and 2022 of eight populations of *Primulina heterotricha* on Hainan Island, south China.

Eight populations roughly covering the whole distribution range of *P. heterotricha* were sampled and included BW (Mt. Bawang), YJ (Mt. Yajia), EX (Mt. Erxian), YG (Mt. Yingge), QX (Mt. Qixian), WZ (Mt. Wuzhi), XA (Xian'an Stone Forest), and JF (Mt. Jianfeng) (Table 1, Figure 2). A total of 176 samples were collected in 2016 and 117 samples in 2022 (Table 1). Although we did not sample prior to 1994 when the Daguangba Reservoir on the Changhua River was completed, sampling from the eight populations before (2016) and after (2022) the construction of the two expressways (expressways 1 and 2 were completed in 2018 and 2019, respectively) would allow us to detect possible genetic and phylogeographic effects of habitat fragmentation. The distance between populations ranged from 6 to 72 km, and the elevation of collected populations varied from 600 (YG) to 1163 m (YJ) above sea level (Table 1). Considering the physical barriers posed by the two expressways and the dam, the populations can be tentatively divided into four groups: the northwest (NW) group of populations (i.e., BW, YJ, EX, and YG), the southeast (SE) group (QX and WZ), the southern (S) group (XA), and the southwest (SW) group (JF). In each population, fresh leaf samples were collected from individuals at least 10 m apart. Each sampled leaf was dried quickly in a separate plastic bag containing 20 to 30 g of silica gel and stored at −80°C later.

**TABLE 1:** Basic information of eight studied populations of *Primulina heterotricha* on Hainan Island.

Sampling site	Population code	Voucher number	Elevation (m)	Sampling size	
				Year 2016	Year 2022
Mt. Bawang	BW	Ling20160803PH003	1011	16	16
Mt. Yajia	YJ	Ling20160807PH011	1163	16	15
Mt. Exian	EX	Ling20160807PH019	920	31	16
Mt. Yingge	YG	Ling20160809PH005	600	9	10
Mt. Qixian	QX	Ling20160809PH013	1000	30	15
Mt. Wuzhi	WZ	Ling20161003PH102	1058	25	15
Mt. Xianan	XA	Ling20161015PH017	783	16	15
Mt. Jianfeng	JF	Ling20161017PH009	918	33	15
Sum				176	117

Total genomic DNA for all samples was extracted using the standard CTAB procedure (Doyle and Doyle 1987) from 30 mg of dried leaf tissue and served as the template for the polymerase chain reaction (PCR). DNA quality and quantity were determined on 0.8% agarose gels stained with 2.5 μL Goldview (Aidlab Biotechnologies Co. Ltd., Beijing, China), with AL2000 DNA marker (Aidlab Biotechnologies).

One nuclear ribosomal DNA (nrDNA) sequence, the ribosomal inter‐transcribed spacer (ITS) region comprising spacer 1, the 5.8S ribosomal gene, and spacer 2 (White et al. 1990), and the two chloroplast DNA (cpDNA) intron‐spacer regions *trn*L‐*trn*F (Taberlet et al. 1991) and *ycf*1b (Dong et al. 2015) were used in this study (Table 2). We are using both nuclear and chloroplast sequences because their different inheritance (biparental for nrDNA and maternal for cpDNA in the majority of angiosperms) facilitates grasping the effects of seed‐ and pollen‐mediated gene flow on genetic structure, while their combined use generally produces stronger phylogenetic inferences. We have added the *ycf*1b to the recurrent combination of ITS/*trn*L‐*trn*F in plant genetics because it is among the most variable regions in plants (Dong et al. 2015).

**TABLE 2:** Primer polymorphic sites (*S*), number of ribotypes/chlorotypes (*Nn*), haplotype diversity (*h*), nucleotide diversity (*π*), and average number of nucleotide differences (*Nd*) for nrDNA and cpDNA amplification and genetic diversity in 2016 and 2022 in *Primulina heterotricha*.

DNA fragment	ITS		*trn*L‐F and *ycf*1b	
	2016	2022	2016	2022
*S*	13	26	48	42
*Nn*	10	18	16	15
*h*	0.809	0.876	0.907	0.874
*π*	0.00435	0.00464	0.00940	0.00868
*Nd*	2.98812	2.91217	12.72468	13.63660
Fragment size	701	640	1405	1592
Tajimas' *D*	0.80474	−1.18345	1.38922	2.25892
Fu's *F*s	1.811	−3.937	12.439	11.3

PCR reactions were set up in a volume of 25 μL, composed of 20 μL ddH_2_O, 2.5 μL 10 × buffer, 0.5 μL 10 mM dNTPs, 0.5 μL each 5 μM primer, 0.5 μL DNA template (about 0.1 ng) and 0.5 μL 5 U/μL Taq polymerase (Aidlab Biotechnologies). The PCR reactions were carried out on a 2720 Thermal Cycler (Applied Biosystems by Life Technologies, Singapore). The PCR program for ITS1/2 and *trn*L‐*trn*F was designed with an initial denaturation of 5 min at 94°C, followed by 35 cycles of 1 min at 94°C, 1 min at 55°C, 1 min at 72°C, and a final extension of 10 min at 72°C. Amplification of *ycf*1b used the following protocol: 4 min at 94°C, 35 cycles of 30 s at 94°C, 40 s at 58°C, and 1 min at 72°C, ending with 10 min at 72°C. All the PCR products were verified based on size by gel electrophoresis. The amplicons were sequenced by an ABI 3730 DNA Analyzer in forward and reverse directions based on the BigDye Terminator Cycle Sequencing Ready Kit (Applied Biosystems, Foster City, CA, USA) in BGI (Beijing Genomics Institute, China).

### 2.2 Genetic Diversity

The chromatograms from both directions of the ITS1/2 and cpDNA sequences were checked visually and edited manually with the software BioEdit (Hall 1999) for base confirmation and contiguous sequence editing; each base/character was equally weighted before analysis, and each indel/gap was represented as a single mutation. Three sequences were manually aligned and trimmed integrally where necessary using MEGA v.6.5 (Kumar et al. 2008) separately. The two non‐coding cpDNA sequences were assembled as a single locus for subsequent analysis by SequenceMatrix v.1.7.8 (Vaidya et al. 2011), and the partition homogeneity test of cpDNA sequences vs. nrITS sequences was carried out with PAUP* v.4.0a164 (Swofford 2002). Since non‐homogeneity of both matrices was detected, nrITS and cpDNA were analyzed independently.

DNASP v.6.12.01 (Rozas et al. 2017) was used to compute the number of identified ribotypes/chlorotypes (*Nn*), haplotype diversity (*h*) within populations, polymorphic sites (*S*), nucleotide diversity (*π*), and the average number of nucleotide differences (*Nd*) separately for nrITS and cpDNA matrices. A Mann–Whitney *U* test (which was performed with “Mann‐Whitney *U* Test Calculator” available from https://www.socscistatistics.com/tests/mannwhitney/) was used to assess the significance of differences in *Nn*, *h*, *S*, and *π* between 2016 and 2022. The geographic distribution maps of ribotypes/chlorotypes at the population level, also separately for nrITS and cpDNA sequences, were constructed and visualized with ArcGIS v.10.8 (ESRI, Redlands, CA, USA).

### 2.3 Phylogenetic Relationships

We inferred the optimal model of nucleotide substitution using MrModeltest v.2.3 (Nylander 2004), based on the AIC (Akaike Information Criteria) (Akaike 1981). The putative most suitable model (GTR + I + G) was used in inferring the phylogenetic relationships of the identified ribotypes, which was done through maximum likelihood (ML) and Bayesian inference (BI) trees (Gao et al. 2015; Genbank with accession number DQ872827).

ML analysis of ribotypes was conducted using MEGA v.6.5 with the optimal substitution model and 1000 bootstrap replicates to assess the support of the resulting groups. BI analysis of ribotypes was conducted using MrBayes v.3.2.6 (nst = 2, rates = equal) (Ronquist et al. 2012), using the optimal model of nucleotide substitutions inferred by AIC in PAUP v.4.0a164. The analysis began with a random tree using Markov Chain Monte Carlo (MCMC) chains with 10 million generations, sampling every 10,000 generations across four independent Bayesian runs. The first 2500 trees (25% of total trees) were discarded as burn‐in, and the remaining trees were summarized in a 50% majority‐rule consensus tree with the posterior probabilities (PP). Chain convergence was assessed by checking the effective sample size that was bigger than 200 for each parameter in Tracer v.1.6 (Rambaut and Drummond 2007), and the length and PP of each branch were visualized by FIGTREE v.1.4.2 (Rambaut 2009).

### 2.4 Population Structure, Mismatch Distribution Analysis, and Neutrality Detection

The population structure of nrDNA and cpDNA sequences was inferred using the Bayesian clustering procedure implemented in STRUCTURE v.2.3.4 (Evanno et al. 2005) without prior structure information; this software identifies the most probable number (*K*) of genetic clusters of origin of the sampled individuals and assigns individuals to clusters. We used MCMC iterations as implemented in STRUCTURE to explore the parameter space considering individual memberships to *K* clusters, ranging from *K* = 1 (null hypothesis of panmixia) to *K* = 8 (the total number of populations sampled). Three independent runs were performed with an admixture model at 10^5^ MCMC iterations and a 10^5^ burn‐in period. The most likely number of groups (*K*, indicating the number of true clusters in the data) and the model values (Δ*K*, according to the second‐order rate of change of cluster *K* that best fits the data) were calculated in STRUCTURE HARVESTER (Earl and vonHoldt 2012). The graphical representation of results was performed in the CLUMPAK server (http://clumpak.tau.ac.il/index.html).

An analysis of molecular variance (AMOVA) was conducted on nrDNA and cpDNA sequences separately to test genetic differentiation within populations, among groups, and populations within groups using GenAlEx v.6.503 (Peakall and Smouse 2012). Population pairwise *F*_ST_ was measured using DNASP v.6.12.01, and population pairwise geographic distances were calculated by GenAlEx. A Mann–Whitney *U* test was also used to determine whether there were significant differences in population pairwise *F*_ST_ values both for nrDNA and cpDNA between 2016 and 2022. To test whether there was local genetic variation attributable to isolation‐by‐distance among populations, the estimates of *F*_ST_/(1−*F*_ST_) and the corresponding natural logarithm of geographic distances (in km) between all pairwise combinations of the eight populations were regressed and subjected to a Mantel test (Mantel 1967) with 999 random permutations in GenAlEx.

The genealogical relationships between ribotypes/chlorotypes based on the Medium‐Joining model were inferred using NETWORK v.4.6.1.0 (http://www.fluxus‐Engineering.com/). To do it, we first saved the sequencing data as.rdf format in DNASP v.6.12.01, then we opened it through the NETWORK software, and finally, we selected the medium‐joining model to output the result. To identify and quantify potential genetic barriers among *P. heterotricha* populations from both nrITS and cpDNA datasets, we calculated the Monmonier's maximum‐difference algorithm in Barrier v.2.2 (Manni et al. 2004). The robustness of these barriers was assessed by bootstrapping genetic distances.

In order to detect possible recent range expansions, Tajima's *D* (Tajima, 1989) and Fu's *F*s (Fu, 1997) were calculated to test the deviations from the null hypothesis of constant population size and neutral evolution for each DNA fragment. Pairwise mismatch distribution and neutrality tests for all populations were conducted in DNASP using the nrITS and cpDNA datasets separately.

## 3 Results

### 3.1 nrITS and cpDNA Datasets

For the samples of 2016, the ITS1/2 sequence matrix comprised 701 bp in total, harbored 13 polymorphic sites (*S*) and 10 ribotypes (*Nn*) from 176 samples (Table 2). At the species level, the ITS1/2 nucleotide diversity (*π*) was 0.00435, the average number of nucleotide differences (*Nd*) was 2.988, and haplotype diversity (*h*) was 0.809 (Table 2). The geographical distribution of ribotypes showed that R1, R5, and R6 occurred in more than one population. R1 ribotype was shared by BW, YJ, EX, and QX, R5 was shared by QX and WZ, and R6 was shared by YG, WZ, and XA. The other seven ribotypes were private (Table 3; Figure 2a).

**TABLE 3:** Nucleotype/haplotype information from nrDNA and cpDNA of eight populations of *Primulina heterotricha* on Hainan Island in 2016 and 2022. Private ribotypes/chlorotypes (occurring in only one population) are given in bold.

Population	2016			2022		
	Type (no. of individuals)	*h*	*Π*	Type (no. of individuals)	*h*	*π*
ITS						
BW	R1 (16)	0	0	R1 (16)	0	0
YJ	R1 (16)	0	0	R1 (12), **R16 (1)**, **R17 (1)**, **R18 (1)**	0.3714	0.00115
EX	R1 (29), **R2 (2)**	0.1247	0.00018	R1 (14), **R2 (2)**	0.2333	0.00464
YG	R6 (1), **R10 (8)**	0.2222	0.00032	R11 (1), **R15 (9)**	0.2000	0.00032
QX	R1 (2), R5 (1), **R8 (27)**	0.1908	0.00064	**R7 (1)**, **R8 (13)**, **R9 (1)**	0.2571	0.00063
WZ	R5 (13), R6 (2), **R7 (10)**	0.5867	0.00285	**R10 (13)**, R11 (1), **R12 (1)**	0.2571	0.00127
XA	R6 (1), **R9 (15)**	0.1250	0.00036	**R13 (14)**, **R14 (1)**	0.3619	0.00464
JF	**R3 (27), R4 (6)**	0.3068	0.00044	**R3 (11), R4 (1)**, **R5 (1)**, **R6 (2)**	0.4667	0.00365
Sum		1.5562	0.00479		2.1475	0.0163
*trn*L‐F and *ycf1*b						
BW	C1 (11), C2 (5)	0.4583	0.00033	C1 (5), **C2 (8)**, C3 (1), **C4 (1)**, **C5 (1)**	0.6833	0.0024
YJ	C1 (9), C2 (1), **C16 (6)**	0.5750	0.00082	C1 (8), C3 (1), **C14 (1)**, **C15 (5)**	0.6381	0.00204
EX	**C4 (15), C5 (2)**, **C6 (5), C7 (6), C8 (3)**	0.7118	0.00380	C1 (15), **C6 (1)**	0.1250	0.00868
YG	**C3 (9)**	0	0	**C13 (10)**	0	0
QX	**C11 (27)**, **C12 (3)**	0.1862	0.00027	**C9 (14)**, **C10 (1)**	0.1333	0.00008
WZ	**C15 (25)**	0	0	**C11 (15)**	0	0
XA	**C13 (10), C14 (6)**	0.5000	0.00074	**C12 (15)**	0	0
JF	**C9 (25), C10 (8)**	0.3788	0.00028	**C7 (14), C8 (1)**	0.1333	0.00008
Sum		2.8101	0.00624		1.713	0.01328

**FIGURE 3:**
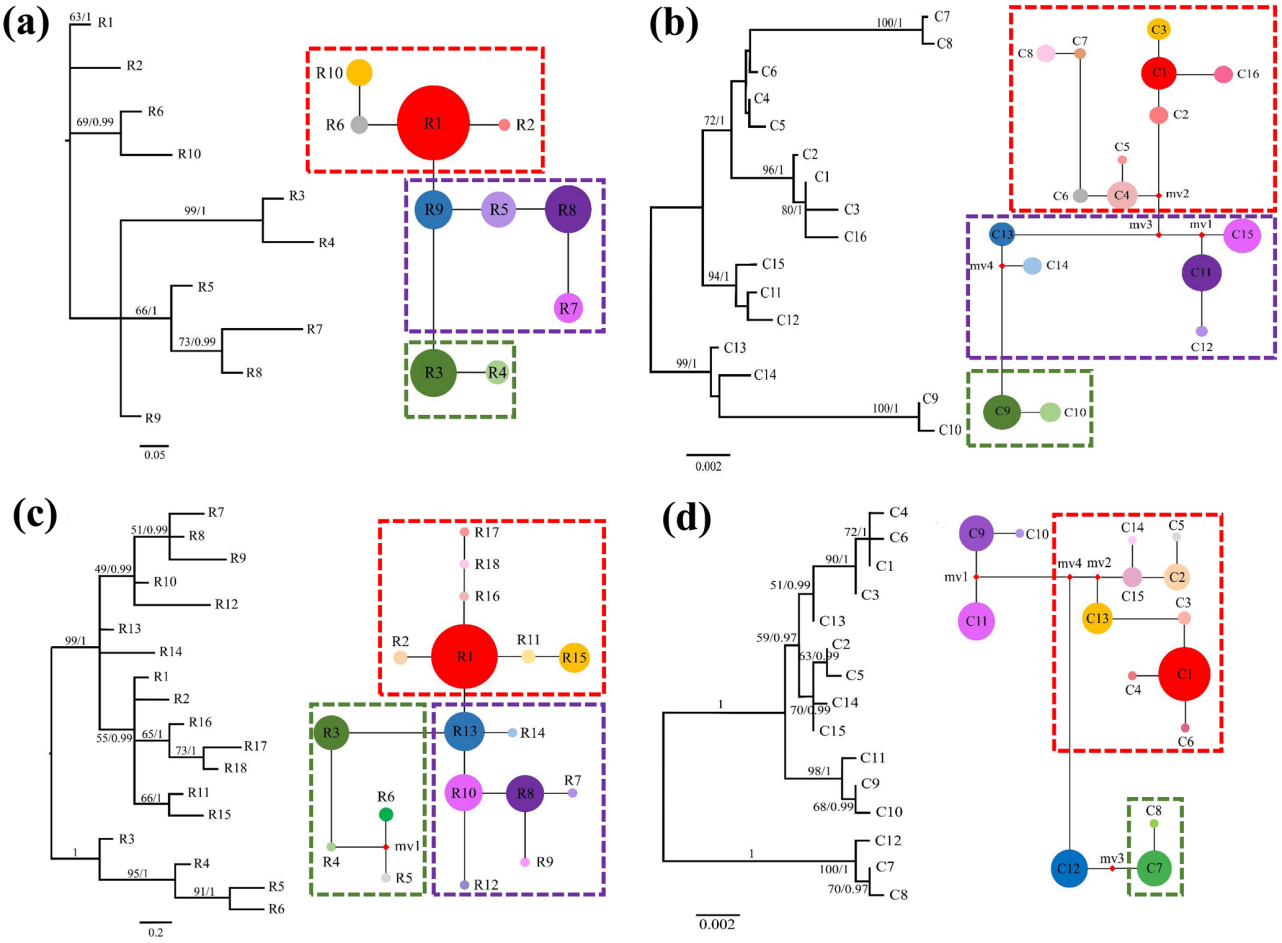
Phylogenetic trees through maximum likelihood (ML) and networks showing genetic relationships among observed ITS ribotypes in (a) 2016 and (c) 2022 and *trn*L‐F and *ycf1b* chlorotypes in (b) 2016 and (d) 2022 of *P. heterotricha* populations. The relative sizes of the circles in the network are proportional to the haplotype frequencies, and missing haplotypes are represented by a small red spot. The frames indicate different clades.

For the 2022 samples, the ITS1/2 sequence matrix comprised 640 bp in total (Table 2). Twenty‐six *S* were present, which allowed the identification of 18 different ribotypes from a size of 117 samples (Table 2). At the species level, *π* was 0.00464, *Nd* was 2.912, and *h* was 0.876 (Table 2). No statistical differences (*p* > 0.05) were found between the 2 years regarding *S*, *Nn*, *h*, and π. Two ribotypes, R1 and R11, were shared by at least two populations (R1 was present in BW, YJ, and EX, and R11 occurred in YG and WZ). The other 16 ribotypes were private (Table 3, Figure 2a). Comparing samples between 2016 and 2022, there was a decrease in both the number (three in 2016 to two in 2022) and the percentage of shared ribotypes (30.0% in 2016 and 11.1% in 2022) (Table 3). Notably, the sharing of ribotypes among the three groups of populations separated by the expressway network (NW, S, and SE) was much diminished; while in 2016 R1 and R6 were shared by NW and SE, and R6 shared by NW, S, and SE, in 2022 only R11 was shared by NW and SE (Figure 3a).

The total length of the combined chloroplast alignments in 2016 was 1405 bp (730 and 674 bp in size for *trn*L‐*trn*F and *ycf*1b, respectively). The alignment contained 48 *S*, and 16 chlorotypes (*Nn*) were present among the 176 samples (Table 2). At the species level, *π* was 0.00940, *Nd* was 12.725, and *h* was 0.907 (Table 2). Of the 16 chlorotypes, only two (C1 and C2) were shared between populations (BW and YJ). The other 14 chlorotypes were private (Table 3, Figure 2b). For the 2022 samples, the combined alignment of two cpDNA regions comprised 1592 bp (870 and 722 bp in size for *trn*L‐*trn*F and *ycf*1b, respectively) which contained 42 *S*, and 15 *Nn* were present among the 117 samples (Table 2). At the species level, *π* was 0.00868, *Nd* was 13.637, and *h* was 0.874 (Table 2). As for the cpDNA, there were no statistical differences (*p* > 0.05) between 2016 and 2022 regarding *S*, *Nn*, *h*, and π. Of the 15 chlorotypes, only two (C1 and C3) were shared (C1 was shared by BW, YJ, and EX, and C3 by BW and YJ) (Table 3). The other 13 chlorotypes were private (Table 3, Figure 2b). In sum, and contrary to the ITS, there were no major differences between the 2016 and 2022 cpDNA datasets, with the same number (two) and almost the same percentage (12.5% vs. 13.3%) of shared chlorotypes, all occurring in the NW group of populations.

### 3.2 Phylogenetic Relationships

For samples of 2016, the network separated the 10 ribotypes of the nuclear ITS into three different monophyletic lineages. Clearly, the ribotypes R1, R2, R6, and R10 that are present in the northwest (NW) group of populations formed a clade; R5 and R7–R9 dominating the south (S) and southeast (SE) populations had the closest phylogenetic relationship to the NW clade, while the R3 and R4 ribotypes that are exclusive to the southwest (SW) populations formed an independent clade from the NW one (Figure 3a). Similarly, for the 2022 samples, the network separated the 18 ribotypes of the nuclear ITS into three monophyletic lineages. The ribotypes R1, R2, R11, R15–R17, and R18, which are dominating the NW populations, converged into a group; R7–R10 and R12–R14 from the S and SE groups showed the closest phylogenetic relationship, and R3–R5 and R6, which are exclusive to the SW group, formed an independent clade (Figure 3c). In addition, BI trees with high BS/PP values based on the ribotypes showed very similar results (Figure S1a,b).

Regarding the cpDNA, the pattern of phylogenetic relationships was very close to that of nrDNA. For both sampling periods (2016 and 2022), the network of chlorotypes showed three monophyletic lineages (Figure 3b,d). The chlorotypes most abundant in the NW group of populations (C1–C8 and C16 in 2016; C1–C6, C13, C14, and C15 in 2022) formed a clade, which was closest to the clade formed by the chlorotypes from the S and SE group of populations (C12–C15 in 2016, C9–C12 in 2022); the clade formed by the chlorotypes exclusive to the SW group (population JF; C9 and C10 in 2016, C7 and C8 in 2022; Figure 3b,d) was the most distant from the NW one. Again, BI trees with high BS/PP values based on chlorotypes showed very similar results (Figure S1c,d).

### 3.3 Population Genetic Structure and Phylogeographical Patterns

The pairwise genetic differentiation (*F*_ST_) was low for the nrITS dataset: for the 2016 samples, *F*_ST_ values ranged from 0 (BW‐EX and YJ‐EX pairs) to 0.010 (JF‐YG and JF‐WZ pairs) with a mean of 0.0045; estimates for the 2022 samples ranged from 0 (BW‐EX pair) to 0.013 (JF‐YG), averaging 0.0054 (Table S1). Regarding the cpDNA dataset, *F*_ST_ values for 2016 samples ranged from 0.001 (BW‐YJ pairs) to 0.020 (JF‐EX and JF‐YG pairs), with a mean of 0.0098, while values for the 2022 samples ranged from 0.001 (JF‐XA pair) to 0.018 (EX‐XA, EX‐JF, OX‐XA, OX‐JF, WZ‐XA, and WZ‐JF pairs), averaging 0.0097 (Table S2). The population separated from the rest by the dam (JF) showed the largest pairwise *F*_ST_ values for both datasets, although for the cpDNA, the population XA also exhibited relatively high levels of genetic divergence (Tables S1 and S2). Indeed, mean *F*_ST_ values between populations belonging to different groups were considerably higher when separated by the dam, that is, JF (= SW region) versus NW region, than when separated by expressways (the rest of pairwise values; Tables S3 and S4); for nrDNA, *F*_ST‐dam[2022]_ = 0.0113 ± 0.0013 and *F*_ST‐dam[2016]_ = 0.0085 ± 0.0010 versus *F*_ST‐expwy[2022]_ = 0.0075 ± 0.0017 and *F*_ST‐expwy[2016]_ = 0.0062 ± 0.0020; for cpDNA, *F*_ST‐dam[2022]_ = 0.0170 ± 0.0008 and *F*_ST‐dam[2016]_ = 0.0193 ± 0.0010 versus *F*_ST‐expwy[2022]_ = 0.0110 ± 0.0077 and *F*_ST‐expwy[2016]_ = 0.0122 ± 0.0030. Thus, as also occurred among individual populations, *F*_ST_ values among groups of populations increased during the period 2016–2022 only for nrITS. As for both the nrDNA and cpDNA datasets, however, there were no statistical differences (*p* > 0.05) in the pairwise *F*_ST_ estimates between 2016 and 2022.

**FIGURE 4:**
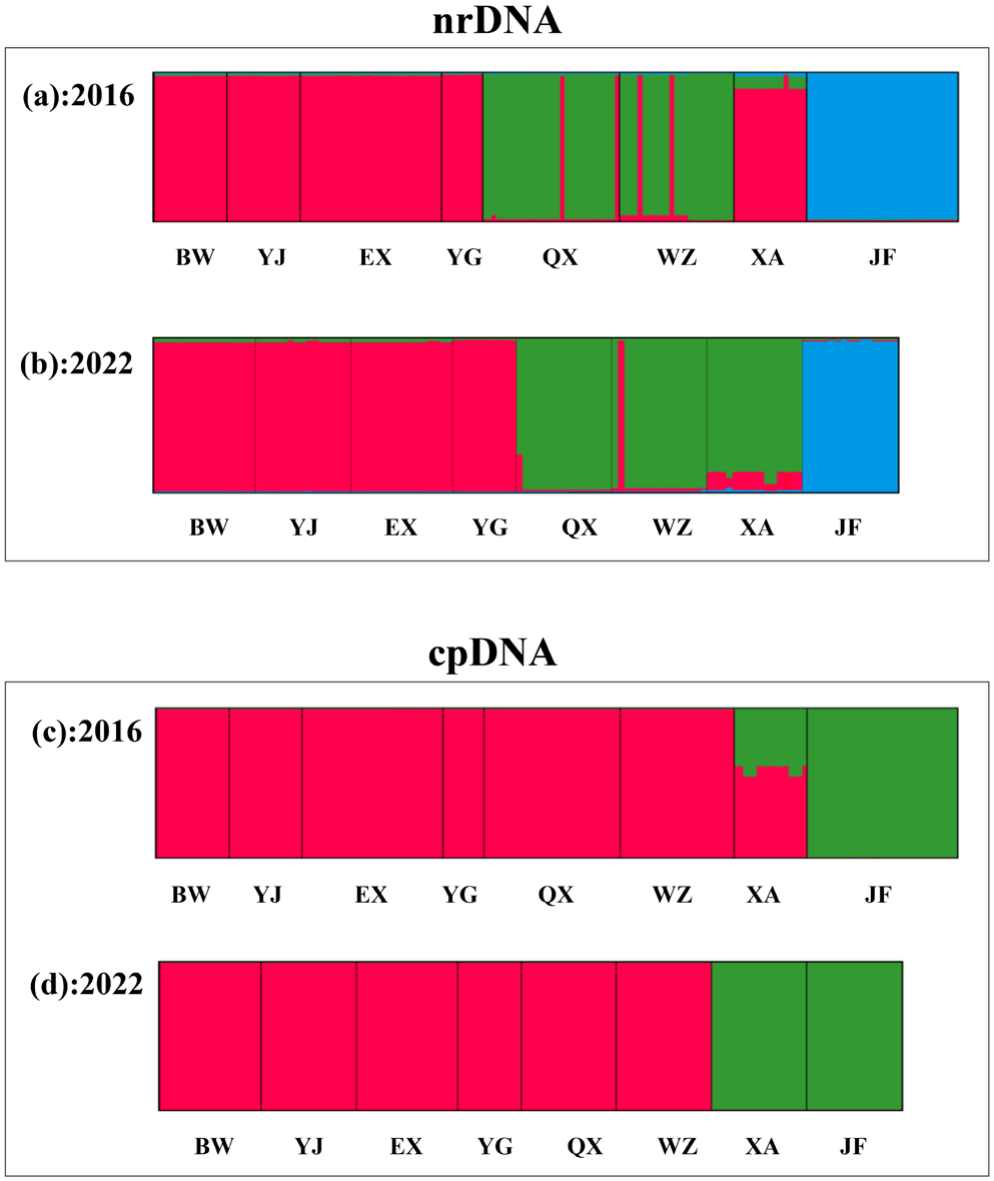
Results of STRUCTURE based on nrDNA and cpDNA samples of *Primulina heterotricha* populations collected in 2016 and 2022.

Based on the Δ*K* approach, *K* = 3 was the most likely number of genetic clusters after running the software STRUCTURE (Figure S1a,b), both for the 2016 and 2022 datasets of nrDNA. In the *K* = 3 grouping scheme, one cluster included the BW, YJ, EX, and YG populations, which represent the NW group of populations (Figure 4a,b). The SE group of populations (QX and WZ) formed another cluster, while the XA population showed two genetic components (NW and SE, with NW rapidly decreasing its weight from 2016 to 2022). The population JF constituted the third cluster.

The Bayesian clustering using cpDNA sequences was somewhat different compared to the nrDNA (Figure 4c,d), as there were only two genetic clusters (*K* = 2) (Figure S1c,d), suggesting that the dam acts as the main genetic barrier in both datasets. The first cluster included all the populations except JF (i.e., group SW) and partially XA (i.e., group S) for the 2016 dataset, while 6 years later the population XA fell within the second cluster.

AMOVA results showed that most ITS genetic variation resides among the four regions (Table 4), although this component declined from 71% (2016) to 62% (2022), and the within‐population component increased from 9% (2016) to 16% (2022). Unlike the nrDNA dataset, the cpDNA genetic variation residing among regions increased from 2016 (72%) to 2022 (90%), while the within‐population component decreased from 10% (2016) to 4% (2022).

**TABLE 4:** AMOVA of genetic variation of eight populations from four regions of *P. heterotricha* based on nrDNA and cpDNA in 2016 and 2022.

	Degrees of freedom	Sum of squares	Estimated variation	Variation (%)
ITS				
Year 2016				
Among regions	3	371.793	2.617	71
Among pops.	4	56.858	0.751	20
Within pops.	168	55.099	0.328	9
Total	175	483.750	3.66	100
Year 2022				
Among regions	3	218.566	2.310	62
Among pops.	4	50.063	0.832	22
Within pops.	109	65.133	0.598	16
Total	116	333.752	3.739	100
cpDNA				
Year 2016				
Among regions	3	1313.175	9.308	72
Among pops.	4	192.434	2.412	18
Within pops.	168	210.613	1.254	10
Total	175	1716.222	12.974	100
Year 2022				
Among regions	3	998.455	12.328	90
Among pops.	4	52.041	0.868	6
Within pops.	109	62.529	0.574	4
Total	116	1113.026	13.770	100

### 3.4 Geographical Barriers, Isolation by Distance, Mismatch Distribution Analysis, and Neutrality Detection

**FIGURE 5:**
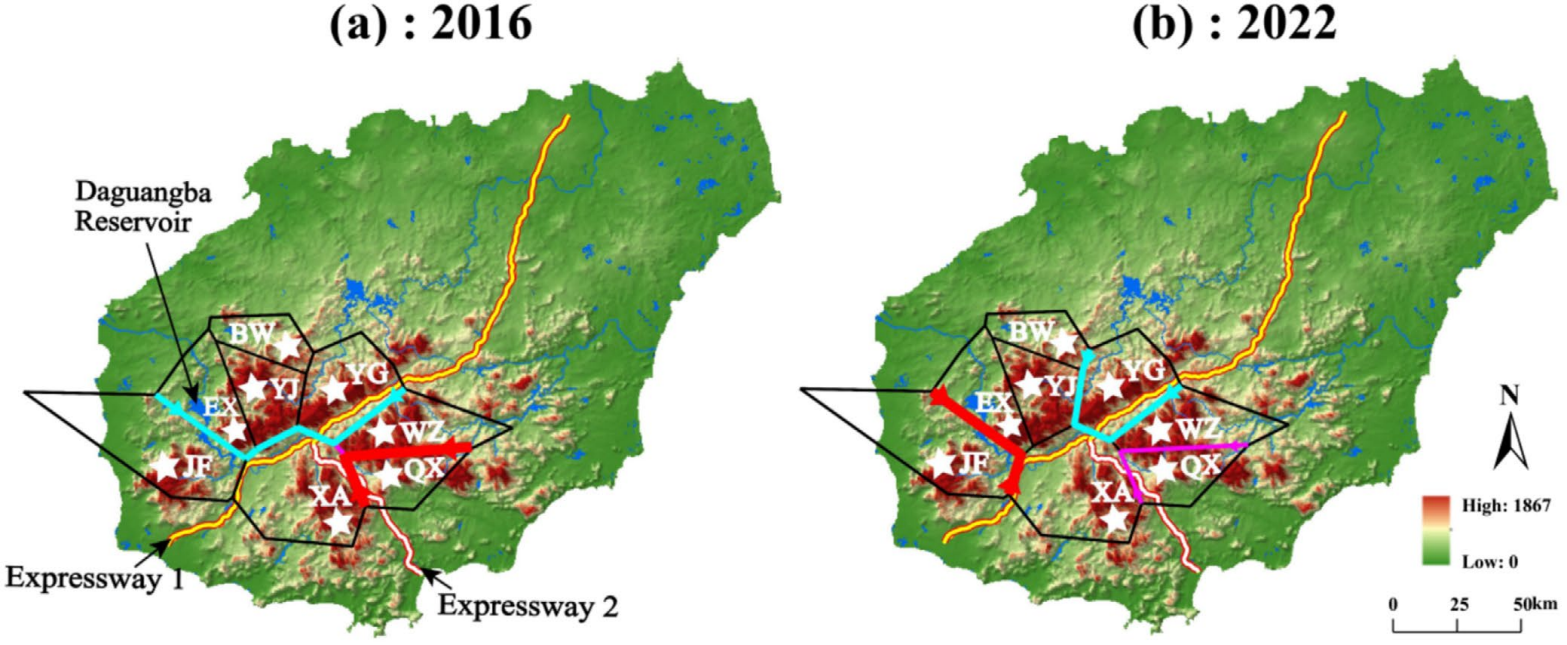
Genetic barriers obtained from BARRIER analysis based on nrDNA in (a) 2016 (Note the third barrier indicated by the faint purple line between XA and WZ) and (b) 2022 of *Primulina heterotricha* populations. Delaunay triangulation is indicated with black lines, while the bold lines (red, blue, and purple lines) indicate the detected genetic barriers.

By calculating Monmonier's maximum difference using the program BARRIER, we detected three geographic barriers using both nrDNA and cpDNA separately, although these barriers slightly differed in position and significance with the marker used and the year assessed. For the 2016 samples, the first barrier using nrDNA (supported by 99% of *F*_ST_ matrices) separated QX from WZ and XA, thus partially corresponding to Expressway 2 (Figure 5a). The second barrier (66% support) separated the NW group from the other populations, thus fitting well with the Daguangba Reservoir and Expressway 1. Finally, the third barrier (33% support) was found between populations XA and WZ (Figure 5a). For the 2022 samples, the first barrier (99% support) separated the JF population from the rest (i.e., the Daguangba Reservoir plus a section of Expressway 1) (Figure 5b). Part of the second (66% support) and third (33% support) barriers could also be assigned to some sections of expressways 1 and 2, respectively (Figure 5b).

**FIGURE 6:**
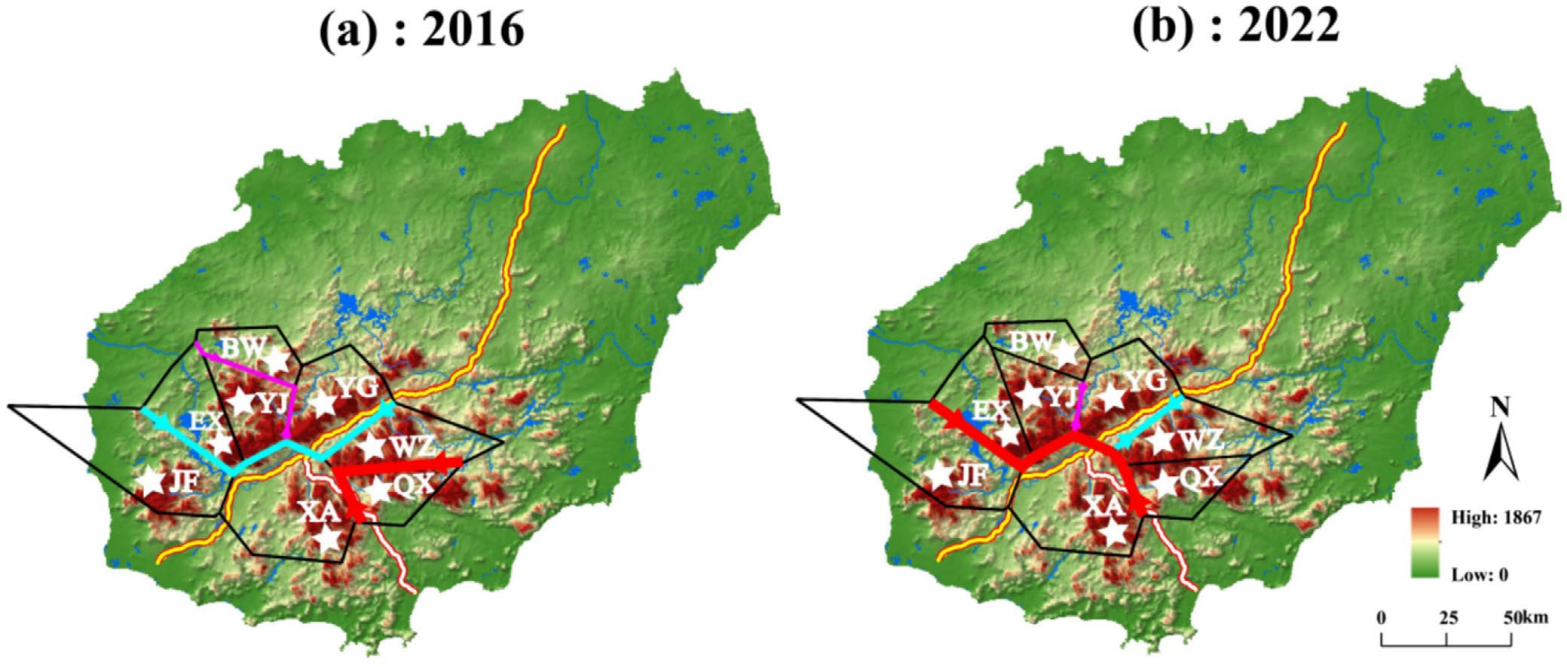
Genetic barriers obtained from BARRIER analysis based on cpDNA in (a) 2016 and (b) 2022 of *P. heterotricha* populations. Delaunay triangulation is indicated with black lines, while the bold lines (red, blue, and purple lines) indicate the detected genetic barriers.

The barrier calculation based on the cpDNA sequence matrix showed similar patterns to those obtained with nrDNA. For the 2016 samples, the only difference was the location of the third barrier, which for the cpDNA separated YJ from BW and YG (Figure 6a). For the 2022 samples, the first barrier changed to be the reservoir but also included some sections of expressways 1 and 2 (Figure 6b). The second barrier corresponded well to the section of Expressway 1 lying between YG and WZ, while the third barrier was located between YJ and YG (Figure 6b).

The Mantel test showed a significant positive correlation between pairwise genetic distance and geographic distance for both nrDNA and cpDNA for the two study periods (Figure S2). The results of Tajima's *D* test and Fu's *F*s test are presented in Table 2 with the associated *p*‐values. The values for *D* and *F*s were positive for the 2016 nrDNA sequences (*D* = 0.80474, *p* > 0.10; *F*s = 1.811), 2016 cpDNA sequences (*D* = 1.38922, *p* > 0.10; *F*s = 12.439), and 2022 cpDNA sequences (*D* = 2.2589, *p* < 0.05; *F*s = 11.3), but were negative for the 2022 nrDNA sequences (*D* = −1.18345, *p* > 0.10; *F*s = −3.937) (Table 2). These results indicate that our sequences (with the only exception of the 2022 cpDNA ones) are in agreement with the null hypothesis of constant population size and neutral evolution. The hierarchical mismatch analysis showed the distributions of differences for all populations (Figure S3), from which the hypothesis of demographic population expansion can be rejected.

## 4 Discussion

### 4.1 Very Quick Effects of Anthropogenic Barriers on Phylogeographical Patterns

The evaluation of the effects of habitat fragmentation on animal and plant species has been traditionally addressed by comparing gene flow, genetic differentiation, and genetic structure of populations in fragmented and non‐fragmented habitats simultaneously (Chung, Nason, et al. 2014; Gao et al. 2015; Schlaepfer et al. 2018). For example, Su et al. (2003) compared the genetic differentiation of populations separated by the Great Wall of China with those not subjected to this huge physical separation for six plants and found attributable differences to the presence of the wall during 600 years. In a second example, the levels of genetic divergence in two crayfishes ( *Faxonius validus* and *F. erichsonianus* ) were significantly higher in streams impounded for 36–104 years than in non‐impounded streams in Alabama, the United States (Barnett et al. 2019). Direct comparisons (i.e., comparing before and after the fragmentation event the populations that have actually been subjected to such disturbance) are probably not available in the literature as it is assumed that the time elapsed from fragmentation should be very long, as enough generations should have passed to observe genetic changes. In their meta‐analysis, Schlaepfer et al. (2018) found that effects are generally only observable after 50 years, although some exceptions could apply, as cases in which only 1–5 generations have passed.

Herein we have measured the fragmentation effects of anthropogenic construction by using the same populations of a plant (*P. heterotricha*) two times within 6 years (i.e., at least two generations of the study plant would have passed). We have detected a significant genetic structure across populations (Figures 3 and 4), and AMOVA shows a high level of genetic variation between regions (Table 4), indicating significant genetic differentiation and limited gene flow among populations and regions. Both the phylogenetic tree and the STRUCTURE results indicate that there are three distinct genetic lineages, corresponding to three clades; i.e., northwest clade (NW region), southwest clade (SW region), and southeast clade (SE region) (Figures 3 and 4). As one may expect for a species inhabiting a rugged mountain area (and where some rivers were dammed since the late 20th century), detectable genetic structure was already present before the expressway construction (2016). Although the effects are not still considerable (probably due to the insufficient time elapsed), we have been able to detect some changes in the genetic structure of *P. heterotricha* just a few years after the two expressways were completed.

The three clades identified for *P. heterotricha* would likely predate the formation of the anthropogenic barriers of the Daguangba Reservoir and Expressways 1 and 2, as the range of this species, which occurs in mountains at relatively high elevations, is naturally fragmented by river valleys. As it can be observed in Figure 2 (and Figures 5 and 6), Expressway 1 has been constructed along the Changhua River (whose valley is, at some parts, up to 3 km wide) that crosses Hainan's south‐central mountain system in a SW–NE direction, and then the river turns into the NW, where it is dammed by the Daguangba Reservoir. Expressway 2 has been built taking advantage of the Tongshi River valley in spite of being much narrower. However, as our results show, the anthropogenic constructions would have intensified the isolation effects of the Changhua River and its associated valley, which has been a geographical barrier since a long time ago (Xiao et al. 2012). In addition, conventional roads were built in these river valleys much earlier; for example, road G224, which was completed in 1954, runs nearly the same route as the two expressways.

The changes in the significance of barriers before and after road construction in the BARRIER analysis (Figures 5 and 6) suggest that geographic isolation caused by human constructions is a key for understanding the present phylogeographical patterns of *P. heterotricha*. Most of the first barrier for both the nrDNA and cpDNA for the year 2016 corresponds to the separation between QX and WZ populations, which does not match any of the anthropogenic constructions. In contrast, for the year 2022, the first barrier coincides with the dam and the expressways for both sequences. Another undisputable signal showing the important role of the anthropogenic barriers on the genetic structure of *P. heterotricha* is the sharp changes in the genetic affinities of population XA, both for nrDNA and cpDNA (Figure 4), which may be caused by the isolation of Expressway 1 but especially Expressway 2. Although it is hard to discern the relative contribution of valleys and rivers on one hand, and expressways on the other hand, the above‐described changes in the genetic patterns from 2016 to 2022 suggest that the two expressways might contribute to shaping the phylogeographical patterns of the island‐endemic *P. heterotricha* just after 6 years of being in place, likely corresponding to two generations of the plant. Geographical discontinuity, including anthropogenic disturbance, is an important factor in population differentiation by weakening or blocking gene flow in many plant species (Slatkin 1985; Su et al. 2003; Kartzinel et al. 2013; Chung, López‐Pujol, et al. 2014; Almeida‐Rocha et al. 2020). These effects are even greater for small herbaceous plants like *P. heterotricha*, with a poor dispersal potential associated with small seeds dispersed largely by water.

### 4.2 Effects of Anthropogenic Barriers on Genetic Structure

Although the different clades of *P. heterotricha* keep stable population dynamics, as revealed by mismatch distribution analysis (Figure S3), the plant would have suffered some detectable effects on its genetic structure, caused by the probably reduced exchange of genes. Notably, the sharing of ribotypes among the three groups of populations separated by the expressway network almost disappeared. Although there were no statistical differences in the pairwise *F*_ST_ estimates between 2016 and 2022, an increased degree both at the population level (*F*_ST[2016]_ = 0.0045, *F*_ST[2022]_ = 0.0054; Table S1) and at the region level (Table S3) was found from 2016 to 2022. In contrast, *F*_ST_ remained invariable along time with cpDNA at the population level (0.0098 in 2016 vs. 0.0097 in 2022; Table S2) and even increased at the group level (*F*_ST_ only decreased between NW and SE regions, which could be attributable to the Expressway 1 construction; Table S4). This incongruence between nrDNA and cpDNA could stem both from the different modes of inheritance of these two markers (nrDNA is biparentally inherited, cpDNA is only maternally inherited) and from the fact that pollen migration rates are often much higher than seed migration rates (Ennos 1994; Petit et al. 2005). Thus, one could expect that the maternally inherited cpDNA (which is only transmitted by seeds) would be much less sensitive to the fragmentation effects, particularly if we take into account that the anthropogenic barriers (particularly the dam) would have affected pollen flow to a much greater extent than seed flow (see below).

Genetic differentiation was higher between populations separated by the dam than between populations separated by the expressways (Tables S3 and S4); i.e., the Daguangba Reservoir showed a more pronounced barrier effect on gene flow. Two factors could explain such an observed pattern. First, the reservoir was built in 1994; therefore, a period of nearly 30 years of isolation would have potentially resulted in more genetic consequences than the newly completed expressways, with a 6‐year period of effects or two generations of *P. heterotricha*. Second, the dam has a water reservoir of about 6 km long and a water surface of 100 km^2^. Thus, the dam probably brings much more barrier effects than the expressways, which are normally only 20–30 m wide. Seeds of *P. heterotricha* are small and may be largely dispersed by raindrops or water courses, while pollination of this species needs small‐sized insects such as *Amegilla leptocoma* and *A. yunnanensis* (Ling et al. 2017a). The seed dispersal mechanism would have been affected by the Daguangba Reservoir, as dams are capable of restricting hydrochory (Andersson et al. 2000). Effects, however, would be much more pervasive regarding pollen dispersal, as the flying distances of these small insects would be hardly enough to connect the two banks of the reservoir (separated by up to 4 km).

Despite having less impact than the reservoir, the effects of expressways are still notable for *P. heterotricha*. In addition to the physical barrier posed by the two high‐capacity roads, the rapid growth of traffic (especially after the end of the COVID‐19 pandemic)—with much more noise and accumulation of pollutants—will increase the levels of disturbance. The fast‐moving traffic and changes in wind conditions associated with expressways probably affected pollinator movements of *P. heterotricha*, thus decreasing pollen flow. Negative effects of roads on the movement (and even survival) of pollinators have been often detected (Stephens et al. 2000; Bhattacharya et al. 2003; Baxter‐Gilbert et al. 2015; Fitch and Vaidya 2021; Dániel‐Ferreira et al. 2022). Although road construction might change hydrological conditions (by affecting natural flow pathways and water quality, e.g., Buchanan et al. 2013), we have not been able to find examples in the literature of roads affecting hydrochory.

In conclusion, we provide evidence that the barrier effects caused by anthropogenic constructions (such as reservoirs and expressways) could produce changes in the genetic structure and phylogeographical patterns within just two generations of this plant. These disruptive effects involving habitat fragmentation may pose primary threats to population regeneration, genetic diversity, and change the species evolutionary processes, especially in endemic, threatened short‐lived plants. To alleviate such negative pressure, we suggest establishing ecological corridors to enhance gene flow between populations separated by these anthropogenic barriers. These could include road tunnels and overpass woodlands, which facilitate the dispersal of plants. Such ecological corridors might also increase the vegetation integrity and habitat continuity between the two sides of these barriers and thus be helpful for the long‐term persistence of the island‐endemic *P. heterotricha* and other rare and endangered species.

## Author Contributions

**Wen‐Ting Jin:** data curation (equal), investigation (lead), methodology (lead), software (lead), writing – original draft (lead). **Shao‐Jun Ling:** data curation (equal), investigation (equal), methodology (equal), visualization (equal), writing – original draft (equal), writing – review and editing (equal). **Myong Gi Chung:** methodology (equal), software (equal), writing – review and editing (equal). **Mi Yoon Chung:** methodology (equal), writing – review and editing (equal). **Jordi López‐Pujol:** formal analysis (equal), methodology (equal), software (equal), writing – review and editing (equal). **Ming‐Xun Ren:** conceptualization (lead), funding acquisition (lead), investigation (equal), methodology (equal), project administration (lead), supervision (lead), writing – review and editing (equal).

## Conflicts of Interest

The authors declare no conflicts of interest.

## Supporting information


Data S1

## Data Availability

All newly acquired sequences have been deposited in GenBank (http://www.ncbi.nlm.nih.gov) under accession numbers MW165394–MW165405 (ITS1/2), MW196628–MW196646 (*trn*L‐*trn*F), MZ391871, MZ479364–MZ479381 (*ycf1*b). The data as supporting material are provided as a file with the name of ‘Supporting Information for review and publication’.
