# Supplementary material for: Human Fragmentation Effects Are Genetically Detectable After 6 Years in an Island‐Endemic Plant
Source: Ecol Evol. 2025 May 12;15(5):e71310. doi: 10.1002/ece3.71310 (PMC12066984; doi:10.1002/ece3.71310)
Supplement: Supplementary file 1 — Data S1 [file ECE3-15-e71310-s001.docx]

**Supporting Information**

**Table S1** Genetic differentiation values (*F*_ST_) of nuclear genes ITS in the year 2016 and year 2022, top right is 2022 and bottom left is 2016.

**Table S2** Genetic differentiation values (*F*_ST_) of chloroplast genes *trn*L-F and *ycf*1b in the year 2016 and year 2022, top right is 2022 and bottom left is 2016.

**Table S3** Regional genetic differentiation values (*F*_ST_, mean±standard deviation) of nuclear genes ITS in the year 2016 and year 2022, top right is 2022 and bottom left is 2016.

**Table S4** Regional genetic differentiation values (*F*_ST_, mean±standard deviation) of nuclear genes *trn*L-F and *ycf*1b in the year 2016 and year 2022, top right is 2022 and bottom left is 2016.

**Figure S1** The number of genetic clusters *K* identified by nrDNA Bayesian inference analysis for 2016 (**a**) and 2022 (**b**), and the most likely number of genetic clusters (*K*) identified by cpDNA Bayesian inference analysis for 2016 (**c**) and 2022 (**d**).

**Figure S2** The results graph of the relationship between genetic and geographic distance for eight populations based on the (**a**) nrDNA in 2016, (**b**) nrDNA in 2022, (**c**) cpDNA in 2016, and (**d**) cpDNA in 2022.

**Figure S3** Mismatch distribution analysis plots across all populations based on nrDNA in 2016 (**a**) and 2022(**b**) and cpDNA in 2016 (**c**) and 2022 (**d**).

| **Table S1** Genetic differentiation values (*F*_ST_) of nuclear genes ITS in the year 2016 and year 2022 between populations of *Primulina heterotricha*; top right is 2022 and bottom left is 2016. | | | | | | | | |
| --- | --- | --- | --- | --- | --- | --- | --- | --- |
| 2022  2016 | BW | YJ | EX | YG | QX | WZ | XA | JF |
| BW | - | 0.001 | 0 | 0.003 | 0.005 | 0.003 | 0.002 | 0.010 |
| YJ | 0 | - | 0.001 | 0.004 | 0.006 | 0.004 | 0.002 | 0.011 |
| EX | 0 | 0 | - | 0.003 | 0.005 | 0.004 | 0.002 | 0.011 |
| YG | 0.003 | 0.003 | 0.003 | - | 0.008 | 0.006 | 0.005 | 0.013 |
| QX | 0.004 | 0.004 | 0.004 | 0.007 | - | 0.003 | 0.004 | 0.012 |
| WZ | 0.005 | 0.005 | 0.005 | 0.007 | 0.003 | - | 0.002 | 0.011 |
| XA | 0.001 | 0.001 | 0.002 | 0.004 | 0.003 | 0.003 | - | 0.009 |
| JF | 0.008 | 0.008 | 0.008 | 0.010 | 0.009 | 0.010 | 0.006 | - |

| **Table S2** Genetic differentiation values (*F*_ST_) of chloroplast genes *trn*L-F and *ycf*1b in the year 2016 and year 2022 between populations of *Primulina heterotricha*; top right is 2022 and bottom left is 2016. | | | | | | | | |
| --- | --- | --- | --- | --- | --- | --- | --- | --- |
| 2022  2016 | BW | YJ | EX | YG | QX | WZ | XA | JF |
| BW | - | 0.002 | 0.003 | 0.002 | 0.006 | 0.006 | 0.017 | 0.017 |
| YJ | 0.001 | - | 0.002 | 0.002 | 0.006 | 0.006 | 0.017 | 0.017 |
| EX | 0.007 | 0.007 | - | 0.003 | 0.007 | 0.007 | 0.018 | 0.018 |
| YG | 0.002 | 0.002 | 0.008 | - | 0.005 | 0.004 | 0.016 | 0.016 |
| QX | 0.007 | 0.008 | 0.007 | 0.009 | - | 0.001 | 0.018 | 0.018 |
| WZ | 0.007 | 0.008 | 0.007 | 0.007 | 0.002 | - | 0.018 | 0.018 |
| XA | 0.011 | 0.012 | 0.011 | 0.011 | 0.008 | 0.008 | - | 0.001 |
| JF | 0.018 | 0.019 | 0.02 | 0.02 | 0.018 | 0.018 | 0.011 | - |

| **Table S3** Regional genetic differentiation values (*F*_ST_, mean±standard deviation) of nuclear genes ITS in the year 2016 and year 2022 between groups of *Primulina heterotricha* populations. SE group include population XA. top right is 2022 and bottom left is 2016. *F*_ST_ value increase for the period 2016–2022 in bold~~;~~ *~~F~~*_~~ST~~_ ~~value decrease for the period 2016–2022 in italics~~. | | | |
| --- | --- | --- | --- |
| 2022  2016 | NW | SE | SW |
| NW | - | **0.0043±0.0019**^expwy^ | **0.0113±0.0013**^dam^ |
| SE | **0.0041±0.0020**^expwy^ | - | **0.0107±0.0015**^expwy^ |
| SW | **0.0085±0.0010**^dam^ | **0.0083±0.0021**^expwy^ | - |

| **Table S4** Regional genetic differentiation values (*F*_ST_, mean±standard deviation) of nuclear genes *trn*L-F and *ycf*1b in the year 2016 and year 2022 between groups of *Primulina heterotricha* populations. SE group include Population XA top right is 2022 and bottom left is 2016. *F*_ST_ value increase for the period 2016–2022 in bold; *F*_ST_ value decrease for the period 2016–2022 in italics. | | | |
| --- | --- | --- | --- |
| 2022  2016 | NW | SE | SW |
| NW | - | **0.0096±0.0056**^expwy^ | *0.0170±0.0008*^dam^ |
| SE | **0.0088±0.0020**^expwy^ | - | *0.0123±0.0098*^expwy^ |
| SW | *0.0193±0.0010*^dam^ | *0.0157±0.0040*^expwy^ | - |

**
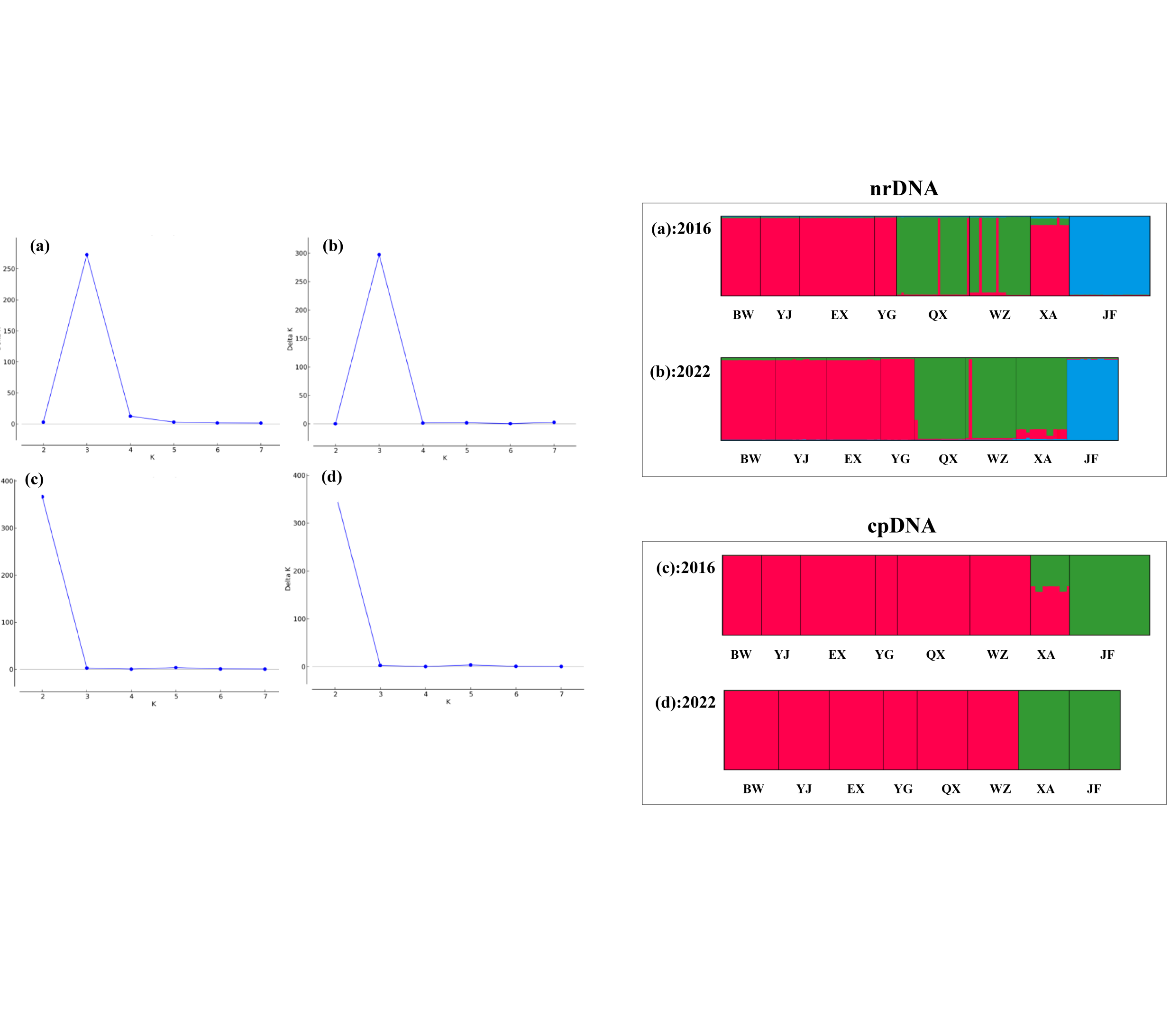
**

**Figure S1** The most likely number of genetic clusters *K* identified by nrDNA Bayesian inference analysis for 2016 (**a**) and 2022 (**b**), and by cpDNA Bayesian inference analysis for 2016 (**c**) and 2022 (**d**) in *Primulina heterotricha*.


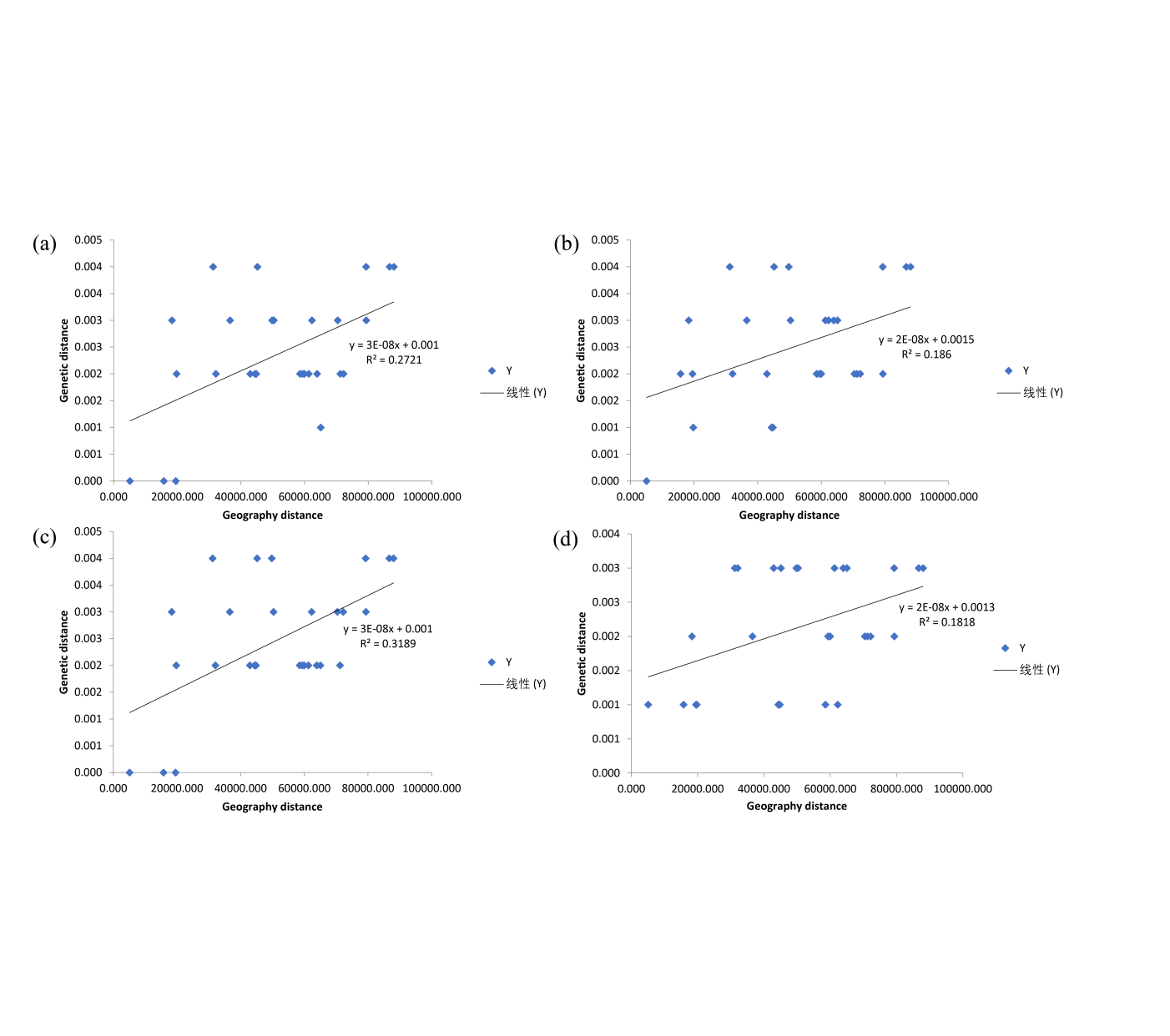


**Figure S2** Graphics showing the relationship between genetic and geographic distance for eight populations of *Primulina heterotricha* based on the (**a**) nrDNA in 2016, (**b**) nrDNA in 2022, (**c**) cpDNA in 2016, and (**d**) cpDNA in 2022.


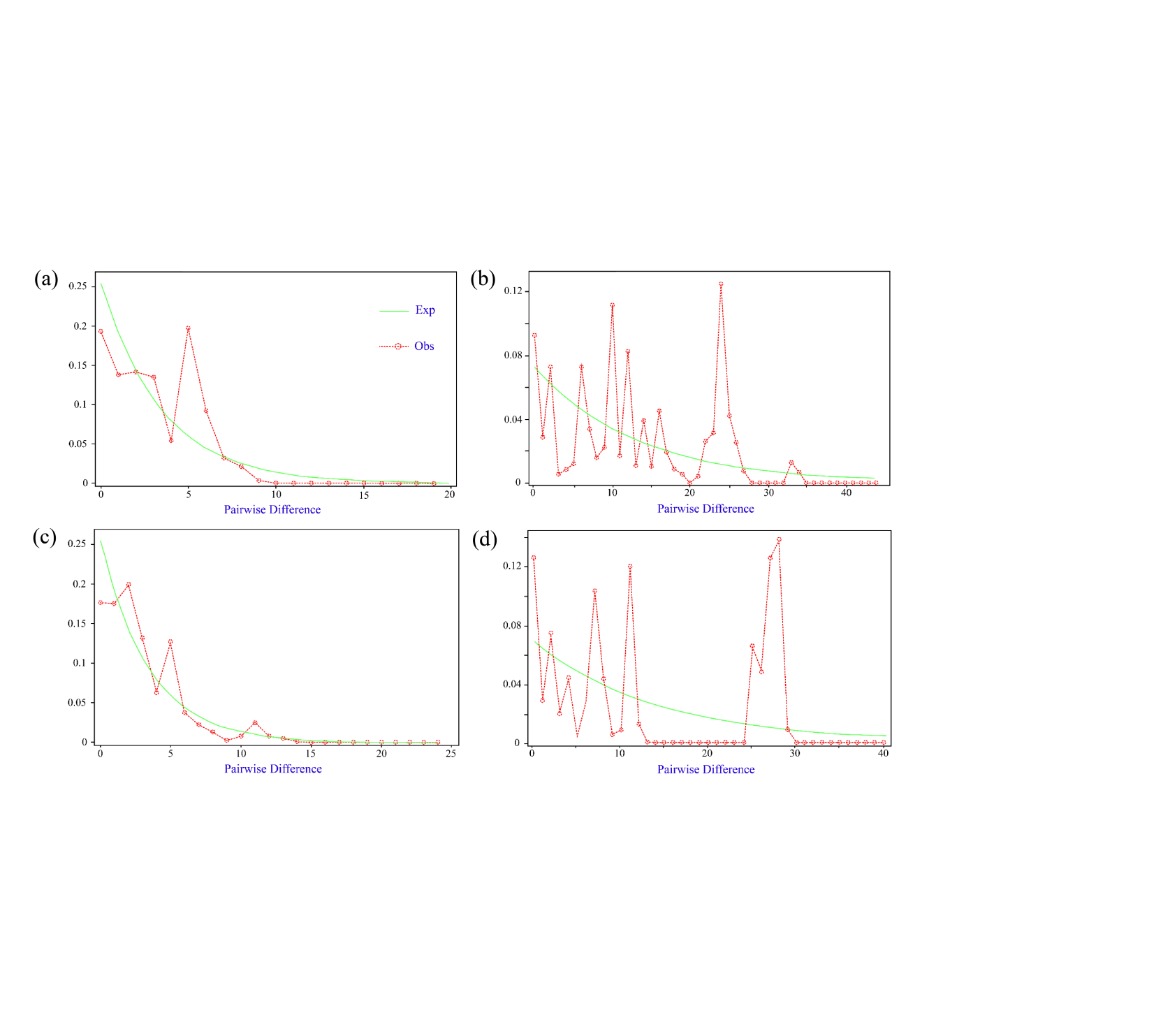


**Figure S3** Mismatch distribution analysis plots across all populations of *Primulina heterotricha* based on nrDNA in 2016 (**a**) and 2022(**b**) and cpDNA in 2016 (**c**) and 2022 (**d**).
